# The Territorial Brigade Camp Hospitals

**Published:** 1909-08-14

**Authors:** 


### The Royal Army Medical Corps Section.

### THE TERRITORIAL BRIGADE CAMP HOSPITALS.

The old Volunteer Force had in its organisation advanced to the stage of being formed into brigades, each of which had its own definite staff, and was made more or less complete within itself. For its medical and surgical needs it had its brigade surgeon, its regimental medical officers and regimental stretcher-bearers; while, in addition, there was attached to it a brigade bearer company, which was part of the R.A.M.C. (V.), and wore its uniform. With the advent of the Territorial Army, and its differently arranged medical service, the brigade surgeon and the brigade bearer company have passed away, and medical assistance is now rendered by (1) an improved regimental system, and (2) by a field ambulance. One of these latter is attached to each brigade, while each of the four regiments forming the brigade has its two medical officers, its stretcher-bearers (bandsmen), its sanitary detachment numbering eight men, and its five water-duty men from the R.A.M.C. (T.F.).

In previous articles the duties of the Territorial regimental surgeon have been fully described both as regards the first-aid and sanitary instruction he is expected to give to his stretcher-bearers, his sanitary detachment, and to some extent to the water-duty men, and also as regards the annual camp. On all these matters full information has been given, and if his unit was to hold a regimental camp of its own he should have no difficulty in providing himself with all the medical and surgical equipment he is entitled to for dealing with the sick and injured during the regiment's stay in camp. When, however, his regiment encamps as part of a brigade, there arises a condition of matters that is somewhat confusing in view of the disappearance of the brigade surgeon of former days and the existence of the field ambulance now attached to each brigade. It is in connection with the latter that the confusion mainly arises, for the conclusion is very naturally come to that its duty is to take care of the sick and injured in camp. This is not the case. A field ambulance belonging to a brigade is, as its name implies, for field work, and its formation and scope of work have been so arranged that its leading features are mobility and its preparedness to go out with its brigade at the shortest notice. This it could not do if its tent division was filled with the sick and injured of a brigade camp. Such a condition of matters would hamper its mobility, and if it did take the field it would do so in a defective and crippled condition, the result of transgressing the fundamental principle that every unit should confine itself to its own duties and not take up those of another unit. If it does so, there follows confusion and inefficiency. In the medical service of the Regular Army, as, for instance, in connection with the expeditionary force, provision has been made for the freeing of the field ambulances of their sick and wounded by the establishment of what are known as "clearing hospitals." Of these we hope to speak in a future article; but it may be mentioned here that as yet they have no place in the Territorial medical service. But the military authorities recognise that even in peace time the exact rôle of the field ambulance must be respected, and that, as they go to the annual camp for acquiring a knowledge of field work, their only opportunity for perfecting themselves in that must not be hampered by throwing on them the care of the sick and injured in brigade camps, and that there must be some provision made for relieving them at once of any casualties that they may have dealt with when out with their brigade on a field day or manœuvres. At places where no military hospital is available, this provision takes the form of a camp hospital, the equipment for which is laid down in Table XI. of the Regulations for the equipment of the Army. This, it must be understood is in excess of, or rather in addition to the regimental equipment drawn by the regimental officer for his unit. Each regiment takes into a brigade camp its own medical and surgical equipment, but in addition to this there is drawn in accordance with Appendix II. Territorial Regulations the equipment of the Brigade Hospital mentioned above. This is done by the brigade-major under brigade arrangements, and on the scale laid down by regulation. Reference to Table XI. shows a detailed list of all the articles furnished, so that there is no need to enumerate them here. It will be seen that there is one circular linen tent to serve as a dispensary, and a small hospital marquee capable of holding ten field-service bedsteads for the accommodation of the sick. All these articles that go to make up the furnishing of the hospital are grouped under the heading of Ordnance equipment, and are indented for by the brigade-major on Army Form G, 968. There is, however, some medical and surgical equipment required, and to understand how this is obtained it is necessary to explain the procedure followed for forming a working staff for this Brigade Hospital. This is done by making the senior of the four regimental surgeons the senior medical officer (S.M.O.) of the camp, and he automatically takes charge of the Brigade Hospital, arranging for its working, and being responsible for the patients in it. Before the assembling of the camp this regimental medical officer should receive notice of his appointment as S.M.O., and it is he who will require to indent for the brigade medical and surgical equipment on Army Form I, 1209, which he will forward properly filled up and signed to the A.M.O. of his Division. The articles allowed are: one medical-companion and water-bottle, four surgical haversacks and water-bottles, one pair of field medical panniers, one No. 1 field fracture box, one stomach pump, and one set of tooth instruments. In addition the Brigade Hospital is also supplied with ten woollen belts, an admission and discharge book (Army Book 27), a diary or ward book (Army Book 39), and with two Mark II. panniers filled with medical comforts; but these last are not applied for by the S.M.O. They come as part of the hospital equipment requisitioned by the brigade-major.

It is upon the S.M.O. that the duty will devolve of making all the necessary arrangements for the carrying on of the work of the Brigade Hospital. To assist him he will have at his disposal the medical officers of the other regiments of the brigade, and a certain number of men to act as nursing orderlies. There is a difficulty as to the source of supply of these latter, as the stretcher-bearers (bandsmen) or the regimental sanitary men are neither available nor qualified, while regulations prevent the employment of paid men from the St. John's or the St. Andrew's Ambulance Associations. Under those circumstances recourse may be had to the water-duty men and a certain number of them detailed for hospital work. A Brigade Hospital being a non-dieted one, patients get their food sent in by their respective companies; but the S.M.O. can always order medical comforts from the special panniers supplied, and, as a rule, in camp no difficulty is experienced in arranging for soup or other extras being obtained from the officers' mess of a patient's regiment. At the end of the camp the S.M.O. must take care that all his equipment is safely packed and returned with the vouchers signed. Seeing that an ambulance wagon is usually furnished to each camp, and that regimental medical officers can replenish at the Brigade Hospital any medical stores that run short, all the requirements of the sick and injured in a brigade camp seem amply provided for.

